# Renal Insufficiency Increases the Combined Risk of Left Ventricular Hypertrophy and Dysfunction in Patients at High Risk of Cardiovascular Diseases

**DOI:** 10.3390/jcm12051818

**Published:** 2023-02-24

**Authors:** Xiaozhao Lu, Qiang Li, Jingru Deng, Yu Kang, Guoxiao Liang, Linxiao Deng, Lei Guo, Haodong Ruan, Zibi Peng, Jiaxi Li, Ning Tan, Jiyan Chen, Jin Liu, Amanda Y. Wang, Yong Liu

**Affiliations:** 1 Department of Cardiology, Guangdong Provincial People’s Hospital (Guangdong Academy of Medical Sciences), Southern Medical University, Guangzhou 510080, China; 2 Guangdong Provincial Key Laboratory of Coronary Heart Disease Prevention, Guangdong Cardiovascular Institute, Guangdong Provincial People’s Hospital (Guangdong Academy of Medical Sciences), Guangzhou 510080, China; 3 The First Clinical School of Medicine, Guangdong Medical University, Zhanjiang 524000, China; 4 The School of Pharmacy, Guangdong Medical University, Dongguan 523000, China; 5 The Faculty of Medicine and Health Sciences, Macquarie University, Sydney, NSW 2109, Australia; 6 The Renal and Metabolic Division, George Institute for Global Health, The University of New South Wales, Sydney, NSW 2042, Australia; 7 Department of Renal Medicine, Concord Clinical School, The University of Sydney, Sydney, NSW 2006, Australia

**Keywords:** renal function, echocardiography, left ventricular function, left ventricular hypertrophy

## Abstract

Background: The identification of asymptomatic structural and functional cardiac abnormalities can help us to recognize early and intervene in patients at pre-heart failure (HF). However, few studies have adequately evaluated the associations of renal function and left ventricular (LV) structure and function in patients at high risk of cardiovascular diseases (CVD). Methods: Patients undergoing coronary angiography and/or percutaneous coronary interventions were enrolled from the Cardiorenal ImprovemeNt II (CIN-II) cohort study, and their echocardiography and renal function were assessed at admission. Patients were divided into five groups according to their estimated glomerular filtration rate (eGFR). Our outcomes were LV hypertrophy and LV systolic and diastolic dysfunction. Multivariable logistic regression analyses were conducted to investigate the associations of eGFR with LV hypertrophy and LV systolic and diastolic dysfunction. Results: A total of 5610 patients (mean age: 61.6 ± 10.6 years; 27.3% female) were included in the final analysis. The prevalence of LV hypertrophy assessed by echocardiography was 29.0%, 34.8%, 51.9%, 66.7%, and 74.3% for the eGFR categories >90, 61–90, 31–60, 16–30, and ≤15 mL/min per 1.73 m^2^ or for patients needing dialysis, respectively. Multivariate logistic regression analysis showed that subjects with eGFR levels of ≤15 mL/min per 1.73 m2 or needing dialysis (OR: 4.66, 95% CI: 2.96–7.54), as well as those with eGFR levels of 16–30 (OR: 3.87, 95% CI: 2.43–6.24), 31–60 (OR: 2.00, 95% CI: 1.64–2.45), and 61–90 (OR: 1.23, 95% CI: 1.07–1.42), were significantly associated with LV hypertrophy. This reduction in renal function was also significantly associated with LV systolic and diastolic dysfunction (all P for trend <0.001). In addition, a per one unit decrease in eGFR was associated with a 2% heightened combined risk of LV hypertrophy and systolic and diastolic dysfunction. Conclusions: Among patients at high risk of CVD, poor renal function was strongly associated with cardiac structural and functional abnormalities. In addition, the presence or absence of CAD did not change the associations. The results may have implications for the pathophysiology behind cardiorenal syndrome.

## 1. Introduction

Heart failure (HF) is globally recognized as a major public health problem, with increasing prevalence and mortality in developing countries [1]. In the latest guideline for the management of HF, pre-HF is defined as a phase of asymptomatic structural and functional cardiac abnormalities [2]. Early recognition and intervention can delay the development of HF and improve the prognosis of patients with HF. Therefore, the early detection of structural and functional changes may help clinicians to recognize patients at pre-HF earlier.

Prior studies and guidelines suggest that renal function insufficiency is one of the most important risk factors for the progression and poorer prognosis of HF [2,3,4]. The changes in heart structure and function are the key pathophysiological elements of heart failure, which may meanwhile underlie the renal pathology, based on interactions through the sympathetic signaling changing of the renin–angiotensin–aldosterone system (RAAS) [5,6,7]. Previous studies indicated that in the general population or patients with CKD, poor renal function was associated with abnormal LV structure and dysfunction [8,9,10]. However, some studies demonstrated inconsistently negative associations between renal function and LV structure and function among the general population or CKD patients [10,11,12]. Few studies have investigated the associations between the cardiac profile and renal function in patients at high risk of HF or cardiovascular diseases (CVD). Therefore, we design this study to systematically examine the associations of renal function with LV structure and systolic diastolic function in high-risk CVD patients.

## 2. Methods

### 2.1. Study Population

This multicenter study cohort was recruited from the Cardiorenal ImprovemeNt II (CIN-II, NCT05050877) study, conducted among five regional central tertiary teaching hospitals in China. Patients who underwent coronary angiography (CAG) and/or percutaneous coronary intervention (PCI) were included. The indications of CAG or PCI were signs or symptoms of ischemia, elevated diagnostic enzymes, or abnormal electrocardiogram findings. All treatments were performed based on the standard clinical practice guidelines [13,14].

**Figure 1:**
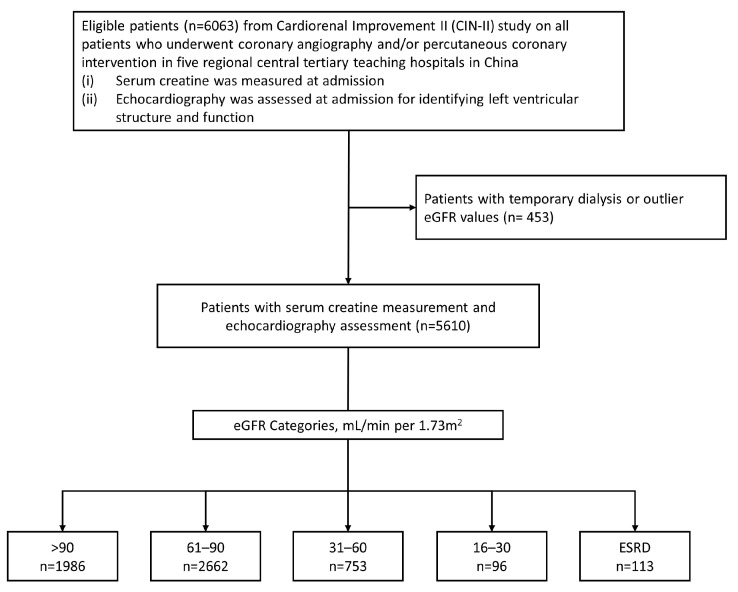
Patient flow diagram.

We enrolled patients (≥18 years) who underwent echocardiographic assessment (structure, systolic, and diastolic function) and had measurements of serum creatinine, height, and weight on admission. Patients with temporary dialysis or outlier eGFR values (>120 mL/min per 1.73 m^2^) were excluded. Finally, 5610 patients were included in this study (Figure 1). The study was approved by the Ethics Committee of Guangdong Provincial People’s Hospital (No.GDREC2019555H[R1]) and conducted in accordance with the principles of the Declaration of Helsinki. All participating sites received institutional review board approval from their own ethics committees.

### 2.2. Data Collection and Definitions

All clinical data of the enrolled patients were collected from the electronic medical record system for all the participant hospitals, including demographic characteristics, medical history, procedures, laboratory examinations, echocardiographic data, and discharge medications. The eGFR was calculated using the Chronic Kidney Diseases Epidemiology Collaboration equation [15]. CKD was defined as eGFR < 60 mL/min per 1.73 m^2^ and end-stage renal disease (ESRD) was defined as eGFR < 15 mL/min per 1.73 m^2^ or the maintenance of dialysis [16,17]. Congestive heart failure (CHF) was defined as New York Heart Association (NYHA) functional class > 2 or Killip class > 1 [18].

### 2.3. Echocardiography Assessment

Echocardiography was performed by the same team of trained cardiac ultrasound doctors at Guangdong Provincial People’s Hospital for all the patients at the time of admission using Philips EPIQ5. The structural indices assessed on echocardiography included the left ventricular (LV) thickness (interventricular septal wall thickness (IVS), posterior wall thickness (PWT), and relative wall thickness (RWT)), LV size (LV end-systolic diameter (LVESD) and LV end-diastolic diameter (LVEDD)), LV systolic function (LV ejection fraction (LVEF)), and LV diastolic function (early mitral inflow peak velocity (E), early mitral annulus TDI velocity (e’), peak velocity flow in the early to late diastole (E/A)). The LV mass was calculated using the linear method and indexed to the body surface area as the LV mass index (LVMI). The RWT was calculated using the formula (2 × diastolic PWT)/LVEDD and was considered to be increased if the result was >0.42. LV hypertrophy was defined as LVMI > 115 g/m^2^ in men and LVMI > 95 g/m^2^ in women. The LV geometry was classified using the LVMI and RWT as normal (no LV hypertrophy and normal RWT), concentric remodeling (no LV hypertrophy and increased RWT), concentric hypertrophy (LV hypertrophy and increased RWT), and eccentric hypertrophy (LV hypertrophy and normal RWT) [19]. LV systolic dysfunction was defined as LVEF < 55%, and LV diastolic dysfunction was defined as E/e’ > 14 [20].

### 2.4. Statistical Analysis

The patients were divided into five groups according to their eGFR levels (>90, 61–90, 31–60, 16–30, and ≤15 mL/min per 1.73 m^2^ or the need for dialysis). Data were presented as the mean with standard deviation (SD) or median with interquartile range (IQR) for continuous variables and as the quantity and frequency (%) for categorical variables. The categorical variables were compared using Pearson’s chi-squared test, and the continuous variables were compared using t-test. Univariable and multivariable logistic regression was used to test the associations between the eGFR categories and LV hypertrophy, as well as LV systolic and diastolic dysfunction. A linear trend test was applied using 5 groups as a continuous variable by assigning the median value of each group to the variable. Restricted cubic splines were plotted to reveal the potential linear associations between the eGFR as a continuous variable and the odds ratio (OR) of LV hypertrophy and LV systolic and diastolic dysfunction. Model 1 was unadjusted, Model 2 was adjusted for age, gender, and body mass index, and Model 3 was adjusted according to Model 2, adding diabetes mellitus (DM), hypertension (HT), CHF, high-density lipoprotein cholesterol, β-blocker, and angiotensin-converting enzyme inhibitor (ACEI) or angiotensin receptor blocker (ARB). All analyses were performed using R software (version 4.2.2; R Foundation for Statistical Computing, Vienna, Austria). A two-sided *p*-value < 0.05 indicated significance for all the analyses.

## 3. Results

### 3.1. Baseline Characteristics

The baseline characteristics of the study cohort are presented in Table 1. The average age of the 5610 patients was 61.6 ± 10.6 years, and 1535 (27.3%) were female. Of those patients, 17.1% (n = 962) had CKD and 2% (n = 113) had ESRD. In comparison with those who had a higher eGFR, patients with a lower eGFR tended to have a worse cardiovascular risk profile (older, with a higher prevalence of hypertension, diabetes mellitus, CHF, CAD, and stroke). With a lower eGFR, there were progressive decreases in the mean LVEF and increases in the mean echo parameters of the LV thickness (IVS and PWT), LV size (LVEDD and LVESD), and LV diastolic dysfunction (E/e’). There was also a correlation between a lower eGFR and a higher proportion of concentric hypertrophy.

**Figure 2:**
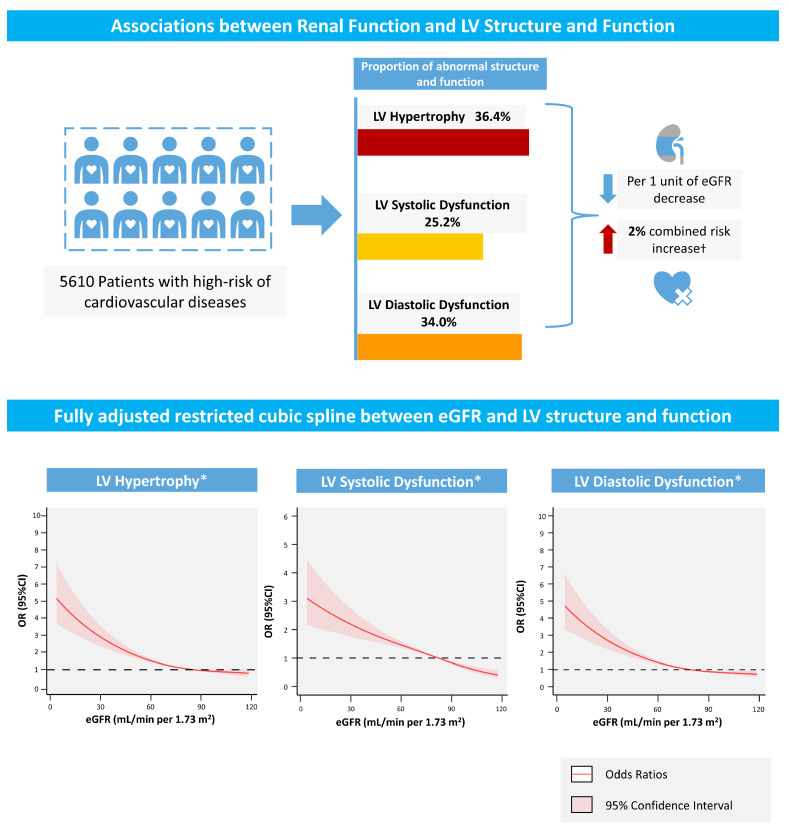
Central Illustration. † All *p* < 0.05. * Adjusted for age, gender, body mass index, diabetes mellitus, hypertension, congestive heart failure, high-density lipoprotein cholesterol, β-blockers, angiotensin-converting enzyme inhibitor/angiotensin receptor blocker.

In total, 36.4%, 25.4%, and 34.0% of the patients had combined LV hypertrophy and LV systolic and diastolic dysfunction, respectively. The associations of the ORs for LV hypertrophy and LV systolic and diastolic dysfunction with eGFR in the fully adjusted restricted cubic spline plots are presented in Figure 2. When fully adjusted for confounders, the associations between eGFR and LV hypertrophy, as well as LV systolic and diastolic dysfunction, remained significant. As depicted in Figure 2, the combined risk of LV hypertrophy and LV systolic and diastolic dysfunction rapidly increases in patients with a lower eGFR.

Subsequently, we ran logistic regression models to evaluate the associations between the eGFR categories and LV hypertrophy, as well as LV systolic and diastolic dysfunction. In the multivariate logistic regression analysis, patients with a lower eGFR were still significantly associated with a higher risk of LV hypertrophy and LV systolic and diastolic dysfunction (Table 2), and a per one unit decrease in eGFR was associated with a 2% heightened risk of combining with LV hypertrophy and systolic and diastolic dysfunction. Compared with the reference of eGFR > 90 mL/min per 1.73 m^2^, patients in the four groups based on the eGFR categories of 61–90, 31–60, 16–30, and ESRD had a higher risk of LV hypertrophy (OR: 1.23; 95% CI: 1.07–1.42, OR: 2.00; 95% CI: 1.64–2.45, OR: 3.87; 95% CI: 2.43–6.24, and OR: 4.66; 95% CI: 2.96–7.54, respectively, P for trend <0.001). Meanwhile, the same increased risk of LV systolic and diastolic dysfunction was observed in the unadjusted and multi-variables adjusted models (all P for trend <0.001).

### 3.2. Subgroup Analysis

The subgroup analyses were consistent with the primary results (Table 3). When the analysis was stratified according to the coronary artery disease (CAD) status, the associations of the eGFR with LV hypertrophy and LV systolic and diastolic dysfunction did not differ significantly among individuals stratified by CAD without interaction (P for interaction: 0.519, 0.348, and 0.779, respectively).

## 4. Discussion

To our knowledge, this is the first study to systematically evaluate the association between renal function and LV structure and systolic and diastolic function among patients at high risk of CVD. Among these patients, more than 1/3 had combined LV hypertrophy, of which concentric hypertrophy formed the highest proportion among the abnormal LV geometries. Almost 1/4 and 1/3 of the patients combined systolic and diastolic dysfunction, respectively. The proportion of LV hypertrophy and systolic and diastolic dysfunction increases directly to the renal function, and a per one unit decrease in eGFR was associated with a 2% heightened combined risk of LV hypertrophy and systolic and diastolic dysfunction.

LV hypertrophy is considered a key pathophysiological feature of patients at pre-HF and a strong predictor of poor prognosis among the general population [2,21]. In our study, 36.4% of patients at high risk of CVD had LV hypertrophy, and 30.8% had concentric hypertrophy. Among patients with CKD stages 3–5 of the Chronic Renal Insufficiency Cohort, Park M. showed that almost half of 3487 patients were classified as having LV hypertrophy, and concentric hypertrophy formed the highest proportion of cases of abnormal LV geometry [10], which is comparable to our study. Matsushita K. reported that the prevalence of LV hypertrophy was 10.6% among 4175 patients in a cohort from Atherosclerosis Risk in Communities population [8]. In addition, the above and other studies of the CKD cohort showed that renal insufficiency was independently associated with LV hypertrophy, especially in advanced CKD. However, several studies demonstrated no independent relations between lower renal function and abnormal LV structure [11,22,23]. The negative results of these studies should be interpreted as aimed at patients with a low risk of a poor cardiovascular profile due to the restricted enrollment of patients with cardiac conditions. In our study, concerning the patients at high risk of CVD, poor renal function was independently associated with LV hypertrophy after adjusting the confounders. Although no strong associations were observed in the relation of renal function and LV hypertrophy in the patients stratified by CAD, it should be noted that a lower eGFR is an independent risk factor for abnormal LV structure, which may require attention in clinical practice.

Systolic and diastolic dysfunction denotes the subsequent changes in LV remodeling and are important predictors for HF and poor prognosis. Previous studies have been inconsistent in determining the association between eGFR and LV systolic and diastolic function, regardless of whether they examined general patients, patients with CKD, or patients with DM [10,12,24]. In addition, strong associations related to LV dysfunction were observed more frequently in patients with advanced CKD. All these studies enrolled patients at a lower risk of CVD than those in our study. Previous studies showed the high prevalence of abnormal LV structure and function in patients with CVD and demonstrated that left ventricular hypertrophy was reversible, whereas diastolic dysfunction was difficult to be improved in patients at high risk of CVD [25,26]. Our study highlighted the high prevalence of LV systolic and diastolic dysfunction in patients at high risk of CVD and showed that even a mild eGFR reduction was consistently associated with a higher proportion of LV systolic and diastolic dysfunction.

Renal insufficiency is associated with structural and functional abnormalities based on multiple mechanisms, representing one of the essential risk factors for patients with pre-HF, and is associated with the development and poor prognosis of HF. There are several possible explanations for these cardiac changes with poor renal function. Firstly, poor renal function increases the burden of salt retention and volume overload, which leads to cardiovascular compensatory changes such as LV remodeling, hypertrophy, and even decompensatory alternations in function [5]. Secondly, patients with CKD are more likely to have diabetes or insulin resistance which may aggravate LV hypertrophy and, subsequently, diastolic and systolic dysfunction through the phosphoinositide-3 kinase–AKT pathway [17,27]. Thirdly, fibroblast growth factor-23 (FGF-23) was found to be elevated in the cases of poor renal function, as it can directly upregulate RAAS by inhibiting angiotensin 2 [28]. Additionally, elevations in FGF-23 can lead to 1,25(OH)_2_D deficiency through 1-α-hydroxylase suppression [29]. All of these factors may contribute to accelerated hypertrophy and dysfunction.

This study has clinical significance and several research implications. Our results demonstrated that poor renal function is an independent predictor of LV hypertrophy and dysfunction among patients at high risk of CVD. Poor renal function is considered a major risk factor for HF, and in recent years, the evaluation of renal function has received more attention from clinicians. However, the assessment of the phenotype of pre-HF and the impact of renal function on the heart are undervalued in clinical practice. This highlights the need for physicians to integrate the early recognition of changes in LV structure and function in clinical routines, especially for patients with mild renal insufficiency. Albuminuria is a well-established risk factor of CVD, and ACEI/ARB use may halt or reverse the progression of albuminuria [30]. In addition, the progression of renal function is significantly associated with increased cardiovascular risk, and multifactorial interventions for risk factors could improve cardiovascular renal outcomes and prognosis. Therefore, early intervention aiming to delay renal function progression or improve the LV structure and function is required in clinical practices [31,32,33,34]. Therefore, future study is needed to verify the efficacy of interventions in protecting renal function upon changes in LV structure and function and their prognostic value for patients with high-risk CVD.

## 5. Limitations

There are several limitations to our study that should be taken into consideration. Firstly, this study was a retrospectively multicenter study, and, thus, our findings reflect statistical associations and do not imply cause–effect relationships. Secondly, our study only used the LVEF and E/e’ for the evaluation of LV systolic and diastolic function, which is one of several parameters used to assess systolic and diastolic function. However, these are easily accessible parameters in clinical practice and can represent the cardiac function of patients. Thirdly, other validated renal function measurements such as the albuminuria level were not incorporated into our study, which could enhance better determination of patients. Fourthly, echocardiography was performed by a team of cardiac ultrasound doctors which may elevate variability and bring an erroneous stratification. Fifthly, the study was aimed at patients with high-risk CVD, who were mainly elderly patients. Although we adjusted for age, the findings should be cautious to extrapolate for other populations. Therefore, more prospective studies are required to evaluate the influence and mechanisms of the relationship between renal function and LV structure and function.

## 6. Conclusions

In summary, a reduction in eGFR was associated with increased LV hypertrophy and reduced systolic and diastolic function among patients at high risk of CVD. In addition, the presence or absence of CAD did not change the outcome. Further studies should be encouraged to explore the underlying risk factors and pathophysiological processes behind cardiorenal syndrome.

## Figures and Tables

**Table 1:** Baseline characteristics of the study population.

		eGFR Categories, mL/min per 1.73 m2					
Characteristics	Overall (N = 5610)	>90 (N = 1986)	61–90 (N = 2662)	31–60 (N = 753)	16–30 (N = 96)	≤15 or Dialysis (N = 113)	*p*-Value
Demographic							
Age, years	61.6 (10.6)	56.2 (9.4)	63.6 (10.0)	67.4 (9.6)	68.6 (9.6)	63.7 (9.6)	<0.001
Female, n (%)	1534 (27.3)	546 (27.5)	699 (26.3)	230 (30.5)	33 (34.4)	26 (23.0)	0.063
Height, cm	162.9 (15.5)	163.4 (16.4)	163.0 (13.9)	161.3 (16.9)	160.7 (18.5)	160.7 (22.8)	0.007
Weight, kg	65.7 (11.8)	66.9 (12.0)	65.4 (11.1)	64.1 (12.4)	62.6 (14.5)	64.6 (13.1)	<0.001
Body mass index, kg/m^2^	24.32 (3.37)	24.56 (3.39)	24.26 (3.24)	24.00 (3.29)	23.75 (5.43)	24.04 (3.80)	<0.001
Medical history							
CAD, n (%)	4254 (75.90)	1433 (72.23)	2029 (76.28)	611 (81.25)	81 (84.38)	100 (88.50)	<0.001
Hypertension, n (%)	3171 (56.6)	938 (47.3)	1508 (56.7)	547 (72.7)	78 (81.3)	100 (88.5)	<0.001
Diabetes mellitus, n (%)	1977 (35.2)	600 (30.2)	890 (33.4)	367 (48.7)	49 (51.0)	71 (62.8)	<0.001
Atrial Fibrillation, n (%)	447 (8.0)	98 (4.9)	229 (8.6)	99 (13.2)	14 (14.6)	7 (6.2)	<0.001
CHF, n (%)	767 (13.7)	170 (8.6)	321 (12.1)	202 (26.9)	25 (26.0)	49 (43.4)	<0.001
Stroke, n (%)	388 (6.9)	91 (4.6)	195 (7.3)	78 (10.4)	11 (11.5)	13 (11.5)	<0.001
Procedure							
PCI, n (%)	3227 (57.5)	1069 (53.8)	1535 (57.7)	475 (63.1)	64 (66.7)	84 (74.3)	<0.001
Laboratory test							
HDL-C, mmol/L	1.0 (0.3)	1.0 (0.3)	1.0 (0.3)	1.0 (0.3)	1.0 (0.3)	0.9 (0.3)	<0.001
LDL-C, mmol/L	2.9 (0.9)	2.9 (0.9)	2.9 (0.9)	2.9 (0.9)	3.0 (1.2)	2.6 (0.8)	<0.001
Echo parameters							
LVEF, %	58.1 (12.8)	60.1 (11.4)	58.1 (13.0)	54.4 (14.4)	52.6 (12.5)	52.6 (13.1)	<0.001
LVEDD, mm	48.7 (8.0)	47.9 (7.1)	48.7 (8.1)	50.2 (8.9)	50.7 (8.6)	52.7 (7.4)	<0.001
LVESD, mm	32.9 (9.6)	31.6 (8.5)	32.9 (9.9)	35.2 (10.8)	35.9 (9.7)	37.6 (8.9)	<0.001
LVRWT, mm	0.4 (0.1)	0.4 (0.1)	0.4 (0.1)	0.4 (0.1)	0.4 (0.1)	0.4 (0.1)	0.484
LVPWT, mm	9.9 (2.1)	9.8 (1.6)	9.9 (2.4)	10.1 (1.7)	10.4 (1.8)	11.2 (2.0)	<0.001
IVS, mm	10.4 (1.9)	10.2 (1.9)	10.3 (1.9)	10.7 (2.0)	11.1 (1.9)	11.8 (1.8)	<0.001
E/A	1.0 (0.5)	1.0 (0.5)	1.0 (0.5)	0.9 (0.6)	1.0 (0.7)	1.0 (0.6)	<0.001
E/e’	14.0 (7.2)	12.6 (5.9)	13.9 (6.5)	16.8 (9.5)	17.2 (7.8)	21.2 (11.7)	<0.001
Left ventricular geometry							<0.001
Normal, n (%)	3153 (56.2)	1265 (63.7)	1526 (57.3)	311 (41.3)	25 (26.0)	26 (23.0)	
Concentric remodeling, n (%)	413 (7.4)	143 (7.2)	209 (7.9)	51 (6.8)	7 (7.3)	3 (2.7)	
Concentric hypertrophy, n (%)	1728 (30.8)	474 (23.9)	796 (29.9)	327 (43.4)	55 (57.3)	76 (67.3)	
Eccentric hypertrophy, n (%)	316 (5.6)	104 (5.2)	131 (4.9)	64 (8.5)	9 (9.4)	8 (7.1)	
Medication							
β-blockers, n (%)	4275 (77.4)	1457 (75.2)	2064 (78.4)	598 (80.0)	66 (70.2)	90 (80.4)	0.013
ACEI/ARB, n (%)	3239 (58.62)	1086 (56.07)	1591 (60.40)	486 (64.97)	34 (36.17)	42 (37.50)	<0.001
CCB, n (%)	1221 (22.1)	377 (19.5)	569 (21.6)	188 (25.1)	39 (41.5)	48 (42.9)	<0.001
Statins, n (%)	4194 (75.9)	1433 (74.0)	2015 (76.5)	589 (78.7)	68 (72.3)	89 (79.5)	0.057

Abbreviation: ACEI/ARB, angiotensin-converting enzyme inhibitor/angiotensin receptor blocker; ACS, acute coronary syndrome; AMI, acute myocardial infarction; CAD, coronary artery diseases; CCB, calcium channel blockers; CHF, congestive heart failure; E, early mitral inflow peak velocity; eʹ, early mitral annulus Doppler tissue imaging velocity; E/A, peak velocity flow in early to late diastole; eGFR, estimated glomerular filtration rate; HDL-C, high-density lipoprotein cholesterol; IVS, interventricular septal wall thickness; LDL-C, low-density lipoprotein cholesterol; LVEDD, left ventricular end-diastolic dimension; LVEF, left ventricular ejection fraction; LVESD, ventricular end-systolic diameter; LVPWT, left ventricular posterior wall thickness; LVRWT, left ventricular relative wall thickness; PCI, percutaneous coronary intervention. Association between eGFR and echocardiographic parameters.

**Table 2:** Associations between the categories of eGFR and LV structure and function in the logistic analysis.

eGFR (mL/min/1.73 m^2^)	Model 1		Model 2		Model 3	
	OR (95% CI)	*p*-Value	OR (95% CI)	*p*-Value	OR (95% CI)	*p*-Value
LV Hypertrophy						
Per 1 unit decrease	1.02 (1.02–1.02)	<0.001	1.02 (1.02–1.03)	<0.001	1.02 (1.01–1.02)	<0.001
>90	Ref	-	Ref	-	Ref	-
61–90	1.30 (1.15–1.48)	<0.001	1.35 (1.18–1.54)	<0.001	1.23(1.07–1.42)	0.005
31–60	2.63 (2.21–3.13)	<0.001	2.71 (2.25–3.26)	<0.001	2.00 (1.64–2.45)	<0.001
16–30	4.87 (3.18–7.61)	<0.001	4.98 (3.21–7.85)	<0.001	3.87 (2.43–6.24)	<0.001
≤15 or dialysis	7.06 (4.63–11.05)	<0.001	7.68 (5.01–12.08)	<0.001	4.66 (2.96–7.54)	<0.001
P for trend	<0.001		<0.001		<0.001	
LV Systolic Dysfunction						
Per 1 unit decrease	1.02 (1.02–1.02)	<0.001	1.02 (1.02–1.03)	<0.001	1.02 (1.01–1.02)	<0.001
>90	Ref	-	Ref	-	Ref	-
61–90	1.39 (1.21–1.61)	<0.001	1.63 (1.40–1.90)	<0.001	1.53 (1.29–1.81)	<0.001
31–60	2.71 (2.25–3.26)	<0.001	3.58 (2.92–4.39)	<0.001	2.67 (2.12–3.37)	<0.001
16–30	3.34 (2.19–5.07)	<0.001	4.62 (2.97–7.41)	<0.001	3.50 (2.11–5.75)	<0.001
≤15 or dialysis	4.22 (2.87–6.21)	<0.001	4.98 (3.35–7.41)	<0.001	2.83 (1.76–4.52)	<0.001
P for trend	<0.001		<0.001		<0.001	
LV Diastolic Dysfunction						
Per 1 unit decrease	1.02 (1.02–1.03)	<0.001	1.02 (1.02–1.02)	<0.001	1.02 (1.01–1.02)	<0.001
>90	Ref	-	Ref	-	Ref	-
61–90	1.49 (1.31–1.70)	<0.001	1.37 (1.19–1.58)	<0.001	1.26 (1.09–1.47)	0.002
31–60	3.25 (2.72–3.87)	<0.001	2.81 (2.32–3.40)	<0.001	2.08 (1.70–2.56)	<0.001
16–30	3.42 (2.26–5.19)	<0.001	2.85 (1.86–4.38)	<0.001	2.30 (1.46–3.65)	<0.001
≤15 or dialysis	11.20 (7.17–18.15)	<0.001	11.01 (7.01–17.94)	<0.001	6.74 (4.16–11.29)	<0.001
P for trend	<0.001		<0.001		<0.001	

Abbreviation: eGFR, estimated glomerular filtration rate; LV, left ventricular; OR, odds ratio; CI, confidence interval. Model 1: unadjusted. Model 2: adjusted for age, gender, and body mass index. Model 3: adjusted for multiple variables (age, gender, body mass index, diabetes mellitus, hypertension, congestive heart failure, high-density lipoprotein cholesterol, β-blockers, angiotensin-converting enzyme inhibitor/angiotensin receptor blocker).

**Table 3:** Subgroup analysis stratified by CAD of the associations between eGFR and LV structure and function.

eGFR (mL/min/1.73 m^2^)	All Patients		CAD		Non-CAD		P for Interaction
	aOR (95% CI)	*p*-Value	aOR (95% CI)	*p*-Value	aOR (95% CI)	*p*-Value	
LV Hypertrophy							
>90	Ref	-	Ref	-	Ref	-	0.519
61–90	1.23(1.07–1.42)	0.005	1.13 (0.95–1.33)	0.167	1.38 (1.04–1.83)	0.027	
31–60	2.00 (1.64–2.45)	<0.001	1.85 (1.47–2.32)	<0.001	2.45 (1.57–3.87)	<0.001	
16–30	3.87 (2.43–6.24)	<0.001	3.48 (2.11–5.84)	<0.001	6.85 (1.92–32.47)	0.006	
≤15 or dialysis	4.66 (2.96–7.54)	<0.001	4.83 (2.98–8.07)	<0.001	3.98 (1.09–18.97)	0.06	
P for trend	<0.001		<0.001		<0.001		
LV Systolic Dysfunction							
>90	Ref	-	Ref	-	Ref	-	0.348
61–90	1.53 (1.29–1.81)	<0.001	1.40 (1.16–1.70)	0.001	2.02 (1.37–3.01)	<0.001	
31–60	2.67 (2.12–3.37)	<0.001	2.30 (1.77–2.97)	<0.001	4.96 (2.82–8.74)	<0.001	
16–30	3.50 (2.11--5.75)	<0.001	2.90 (1.67–4.98)	<0.001	5.84 (1.46–21.38)	0.009	
≤15 or dialysis	2.83 (1.76–4.52)	<0.001	2.62 (1.59–4.29)	<0.001	3.14 (0.62–13.01)	0.133	
P for trend	<0.001		<0.001		<0.001		
LV Diastolic Dysfunction							
>90	Ref	-	Ref	-	Ref	-	0.779
61–90	1.26 (1.09–1.47)	0.002	1.14 (0.96–1.36)	0.132	1.54 (1.15–2.06)	0.004	
31–60	2.08 (1.70–2.56)	<0.001	1.76 (1.39–2.22)	<0.001	3.12 (2.00–4.91)	<0.001	
16–30	2.30 (1.46–3.65)	<0.001	1.95 (1.18–3.24)	0.010	4.29 (1.37–14.20)	0.013	
≤15 or dialysis	6.74 (4.16–11.29)	<0.001	6.21 (3.49–10.85)	<0.001	7.72 (2.11–36.89)	0.004	
P for trend	<0.001		<0.001		<0.001		

Abbreviation: aOR, adjusted odds ratio; CAD, coronary artery diseases; CI, confidence interval; eGFR, estimated glomerular filtration rate; LV, left ventricular. Multivariable logistic regression was adjusted for multiple variables (age, gender, body mass index, diabetes mellitus, hypertension, congestive heart failure, high-density lipoprotein cholesterol, β-blockers, angiotensin-converting enzyme inhibitor/angiotensin receptor blocker).

## Data Availability

The datasets generated and analyzed during the current study are not publicly available due to the institution’s policy but are available from the corresponding author upon reasonable request.
